# Behavioural evidence for distinct mechanisms related to global and biological motion perception

**DOI:** 10.1016/j.visres.2017.08.004

**Published:** 2018-01

**Authors:** Louisa Miller, Hannah C. Agnew, Karin S. Pilz

**Affiliations:** a Leverhulme Research Centre for Forensic Science, University of Dundee, United Kingdom; b Department of Psychology, University of Aberdeen, United Kingdom

**Keywords:** Motion perception, Global motion, Motion coherence, Biological motion

## Abstract

The perception of human motion is a vital ability in our daily lives. Human movement recognition is often studied using point-light stimuli in which dots represent the joints of a moving person. Depending on task and stimulus, the local motion of the single dots, and the global form of the stimulus can be used to discriminate point-light stimuli. Previous studies often measured motion coherence for global motion perception and contrasted it with performance in biological motion perception to assess whether difficulties in biological motion processing are related to more general difficulties with motion processing. However, it is so far unknown as to how performance in global motion tasks relates to the ability to use local motion or global form to discriminate point-light stimuli. Here, we investigated this relationship in more detail. In Experiment 1, we measured participants’ ability to discriminate the facing direction of point-light stimuli that contained primarily local motion, global form, or both. In Experiment 2, we embedded point-light stimuli in noise to assess whether previously found relationships in task performance are related to the ability to detect signal in noise. In both experiments, we also assessed motion coherence thresholds from random-dot kinematograms. We found relationships between performances for the different biological motion stimuli, but performance for global and biological motion perception was unrelated. These results are in accordance with previous neuroimaging studies that highlighted distinct areas for global and biological motion perception in the dorsal pathway, and indicate that results regarding the relationship between global motion perception and biological motion perception need to be interpreted with caution.

## 1 Introduction

We constantly perceive movement from the world around us, from leaves being blown by a gust of wind, to people walking in the street. The former is related to bottom up processing and is predominantly stimulus driven: we integrate the motion of all leaves into the percept of their global movement. The latter is an example of biological motion, which requires top-down processing and a reliance on stored movement patterns.

In an experimental setting, random dot kinematograms (RDK) are often used to study the properties of global motion perception. These RDK stimuli resemble a dense swarm of bees, and by integrating the local motion of all ‘bees’, it is possible to determine the general direction in which the swarm is flying. Stimulus parameters are often chosen such that it is impossible to track individual dots and it is necessary to integrate the motion of the individual dots to achieve a global impression of coherent motion. There are a number of factors that affect our ability to determine the general direction of movement, such as the proportion of dots moving in a single direction (coherence, or signal-to-noise ratio), and the duration of the stimulus. The discrimination of global motion is thought to rely on processing in area hMT/V5, as part of the dorsal visual stream. Neurons in this area have been shown to be sensitive to global motion, with a similar sensitivity to behaviourally measured motion coherence thresholds (Britten, Shadlen, Newsome, & Movshon, 1992). In addition, Braddick, O’Brian, Wattam-Bell, Atkinson, and Hartley (2001) found that neurons in hMT/V5 show greater activation to coherent than incoherent global motion, whereas in V1, for example, activation is higher for incoherent motion.

In contrast to global motion perception as described above, biological motion describes the complex visual pattern we perceive as the movement of a person or other animate being. This kind of motion is often investigated experimentally using point-light stimuli (often walkers): simplified dynamic visual representations of the human (or animal) form, in which small dots represent the location of the head and major joints of the body. With just this sparse information, adults can quickly identify human movement (Johansson, 1973). The perception of biological motion is reportedly present from as early as 5 months of age (Bertenthal, Proffitt, & Kramer, 1982), and by adulthood we are able to determine the gender (Kozlowski and Cutting, 1977, Pollick et al., 2005), emotions (Dittrich et al., 1996, Roether et al., 2008, Spencer et al., 2016), and even individual identity of point-light stimuli (Kozlowski and Cutting, 1977, Loula et al., 2005, Troje et al., 2005).

It was originally thought that the local motion cues of the single dots were key to biological motion perception. Due to the robustness of biological motion perception from just a few point-lights, Johansson (1973) believed that the process must be driven by low-level processes. Mather, Radford, and West (1992) investigated this idea in a series of experiments. They asked participants to discriminate normal point-light walkers from walkers in which the top and bottom half were moving in opposite directions. In a first experiment, they varied the temporal characteristics of the stimuli and found that participants were only able to discriminate the walkers with short inter-frame intervals. A second experiment showed that participants’ performance was also affected by the amount of spatial displacement of each dot from frame-to-frame. The authors suggested that these results highlight a reliance on low-level motion processes for processing point-light walkers, as such processes are typically implemented over short temporal and spatial increments. Interestingly, when dots were removed from the animations, performance was only significantly affected by the removal of dots representing the wrists and ankles, the dots with the most informative motion trajectories, which led the authors to the overall conclusion that low-level processes appear to be essential for biological motion processing.

Despite evidence of the importance of low-level motion processes for the perception of biological motion, other studies showed that point-light walkers can be discriminated by form information alone. Beintema and Lappe (2002), for example, disrupted the local motion information in point-light walkers by placing dots at random points along a limb in each frame of the motion sequence, rather than on the joint, thereby destroying the local motion trajectories but preserving the global form of the walkers. Similar stimuli have been used many times to show that participants are able to discriminate motion direction and actions from point-light animations even when the local motion information is disrupted (Beintema and Lappe, 2002, Pilz et al., 2010, Lange and Lappe, 2006, Agnew et al., 2016). Disrupting the local information by embedding the walker in noise has also been shown to not significantly affect the perception of point-light walkers (Bertenthal & Pinto, 1994), which indicates that the global form is important for biological motion processing.

More recent research converges on the idea that biological motion can be processed using both the local signals and the global form, and that it depends on the task and specific stimulus used as to which one is more beneficial (Thirkettle, Benton, & Scott-Samuel, 2009). Performance seems to be best when both kinds of information can be accessed. A model by Giese and Poggio (2003) nicely summarises this idea and suggests that biological motion can be processed via motion analysis in the dorsal stream and via form analysis in the ventral stream in a bottom-up manner, with information from both pathways being integrated in higher-level areas. In the dorsal stream, local motion signals are processed in early visual areas such as V1 or V2, and integrated into more complex global motion signals in MT/V5. In the ventral pathway, early visual areas process orientation information that is integrated into more complex form features in areas such as V2 or V4 and snapshots of more meaningful shapes in IT, for example. The information from both pathways is then integrated over time into meaningful biological motion in the superior temporal sulcus, for example. Compelling evidence for the dual stream hypothesis was provided by Mather, Battaglini, and Campana (2016) who used TMS over hMT/V5 while participants performed a coherent motion and a biological motion direction discrimination task. Whereas TMS disrupted the processing of coherent motion, biological motion perception remained unaffected. These results clearly highlight that hMT/V5 is not necessary for processing biological motion.

As indicated above, many studies have investigated the contribution of local motion and global form to biological motion processing. Performance in tasks involving more basic global motion processing such as the discrimination of motion direction from RDKs is often compared to performance in biological motion perception in special populations such as schizophrenia, autism or ageing, to assess whether deficits in biological motion perception are related to a more general motion processing deficit (e.g., Spencer et al., 2000, Billino et al., 2008, Koldewyn et al., 2010, Spencer et al., 2013). However, it is unclear up to now as to whether these two abilities are related and whether it is reasonable to make such a comparison. Therefore, this study directly investigates the relationship between global motion perception and local motion and global form processing in biological motion perception. In Experiment 1, we measured motion coherence thresholds for translational motion using RDKs, and participants were asked to discriminate the facing direction of point-light actions that contained primarily local motion information, global form information or both. If there was a relationship between processing global motion and the local motion information in point-light stimuli, we would expect a strong correlation between motion coherence thresholds and the ability to discriminate actions that primarily contained the local motion information. Experiment 2 assessed the relationship between biological motion direction discrimination with or without noise, and coherent motion perception from RDKs. A correlation between motion coherence thresholds and the ability to discriminate point-light stimuli in noise would indicate that both tasks rely on the ability to discriminate signal from noise.

## 2 Experiment 1

### 2.1 Methods

#### 2.1.1 Subjects

Participants were recruited from the staff and student population at the University of Aberdeen. Twenty-one individuals (5 males), aged 18–29 (M = 22.71, SD = 2.97) participated. All had normal or corrected to normal vision (visual acuity >0.8 on the ETDRS chart). Participants received £5/hour for their participation and all gave written informed consent. The experiment was carried out in accordance with the Declaration of Helsinki.

#### 2.1.2 Apparatus

Stimuli were presented on a 19 inch CRT Dell monitor (model M993S) with a resolution of 1024 × 768 pixels and a refresh rate of 100 Hz. Stimuli were presented using the MATLAB (The MathWorks, Inc., Natick, MA, USA) based Psychtoolbox extension (Brainard, 1997, Kleiner et al., 2007).

#### 2.1.3 Stimuli

##### 2.1.3.1 Motion coherence task

Stimuli were RDKs in a circular aperture of 9.4 deg with 150 dots. These white dots were 2 pixels in area, had a limited lifetime of 200 ms, and were shown on a black background. Dots were randomly positioned within the aperture at the beginning of each trial with a random lifetime. At the end of a dot’s lifetime, or if the dot moved out of the aperture, it was replaced at a random location in the aperture on the next refresh, moving in its previously assigned direction. Motion coherence (the percentage of dots moving in the same direction) was set to 10, 20, 30, 40, 50, 60, and 80. There were 20 trials for each motion direction (left or right) for each level of motion coherence, resulting in 280 trials total. The motion direction of each noise dot was randomly chosen between 0 and 360 degrees. Stimulus duration was set to 400 ms. For each observer, a logistic psychometric function was fit to the data and the coherence at 82.5% performance was determined for each participant individually.

##### 2.1.3.2 Biological motion

**Fig. 1:**
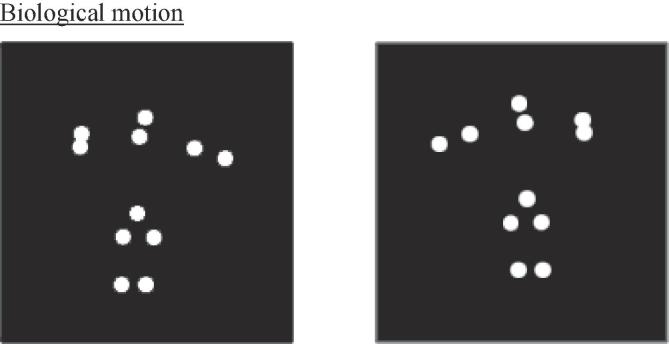
Biological motion stimuli showing a right- and left-facing figure serving a tennis ball.

A point-light animation of a figure serving a tennis ball was used throughout (Vanrie & Verfaillie, 2004). The figure was located in the centre of the screen with a random displacement of up to 10 pixels in any direction from the centre on each trial. Eleven white dots, set against a black background, indicated the position of the head and major joints. The animation showed either a right- or leftward serve (see Fig. 1).

In addition to the ‘normal’ condition, ‘scrambled’ and ‘random’ stimuli were also used, similar to Pilz et al. (2010). Scrambled point-light actions maintained the local direction of movement of all dots, however the starting positions were spatially displaced along the y-axis, disrupting the global form of the figure (see Bertenthal and Pinto, 1994, Thornton et al., 1998 and Troje & Westhoff, 2006). Random-position actions contained the appropriate global form, so the outline of the figure was still visible. However, local information was disrupted by placing the dots on a random position on the adjacent limb at each frame of presentation (see Beintema & Lappe, 2002). Stimuli were presented for 200 ms. Each condition was presented in a separate block. There were 160 trials per block, resulting in a total number of 480 trials.

#### 2.1.4 Procedure

Participants sat 60 cm from the computer monitor in a dimly lit room, with their head stabilised on a chinrest. Task order (RDK/biological motion) and biological motion condition order was counterbalanced, and trials were presented as above.

Participants were instructed to indicate the direction of motion as quickly and accurately as possible using keys on a standard QWERTY keyboard. For each participant, we recorded percent accuracy for each condition.

### 2.2 Results

Low-level motion coherence threshold as measured in the RDK task ranged from 10% to 100% coherence, with a median of 26%.

**Fig. 2:**
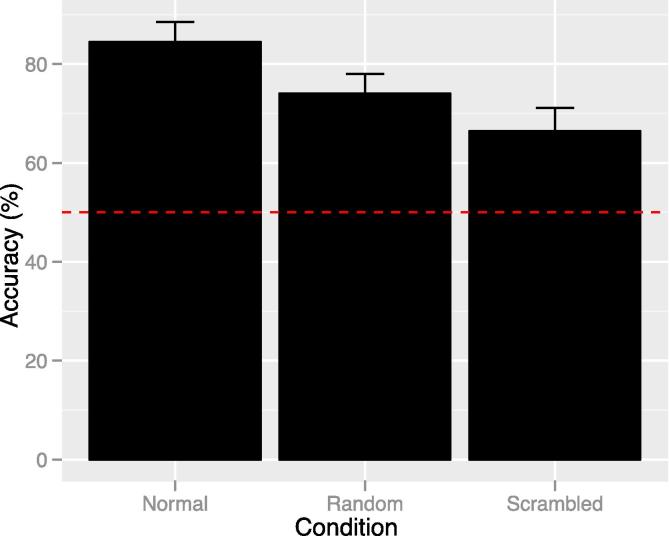
Mean accuracy for each biological motion condition (error bars show SE and red line shows chance level). There was a significant difference in accuracy for normal compared to both random and scrambled stimuli (p < .05).

Mean accuracy in each biological motion condition is shown in Fig. 2. A repeated measures ANOVA on accuracy in each condition (normal, random, scrambled) revealed a significant effect of condition: F(1.549, 30.984) = 8.984, p = .002 (Greenhouse-Geisser (GG) corrected), $\eta_{p}^{2}$ = 0.310. Post hoc comparisons (all Bonferroni corrected) showed that this effect was driven by significantly better accuracy in the normal condition compared to both random and scrambled (mean difference = 10.42, SE = 2.91, t(20) = 3.578, p = .006; and mean difference = 18.04, SE = 4.92, t(20) = 3.667, p = .005 respectively). There was no significant difference between scrambled and random (mean difference = 7.62, SE = 4.70, t(20) = 1.621, p = .362).

**Fig. 3:**
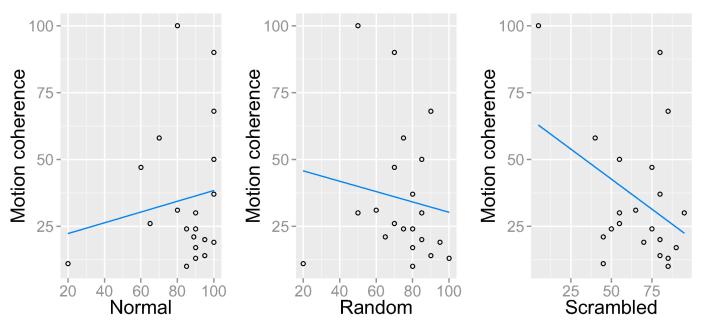
Correlations between percentage accuracy for each biological motion condition and motion coherence (using RDK stimuli).

To investigate a possible relationship between the processing of high- and low-level visual motion, accuracy in the three biological motion conditions and motion coherence threshold in the RDK task were correlated using Spearman correlations (see Fig. 3). Motion coherence threshold in the RDK task was not significantly correlated with accuracy in any of the three biological motion conditions: r_s_ = 0.112, p = .630, N = 21 (normal); r_s_ = −0.255, p = .264, N = 21 (scrambled); and r_s_ = −0.255, p = .264, N = 21 (random), all two-tailed.

**Fig. 4:**
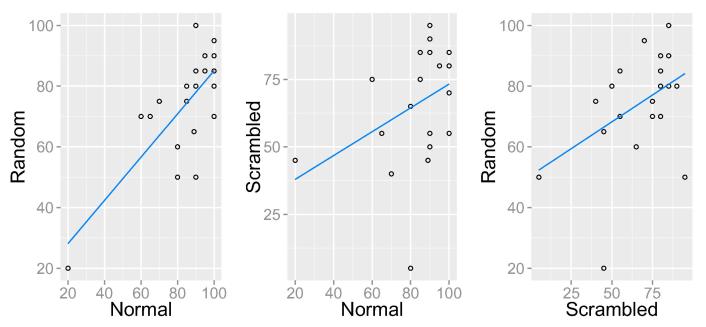
Correlations between percentage accuracy for each biological motion condition. There is a significant correlation between normal and random (left) and normal and scrambled (centre). The correlation between random and scrambled (right) was not significant.

When considering the related nature of the three biological motion tasks (Fig. 4), there was a significant correlation between normal and random (r = 0.655, p < .001, N = 21), and normal and scrambled (r_s_ = 0.450, p = .04, N = 21), however the correlation between random and scrambled was not statistically significant (r_s_ = 0.374, p = .095, N = 21). Controlling for multiple comparisons using Bonferroni correction, only the correlation between random and normal biological motion survived, which indicates that global form information is more relevant for discriminating point-light stimuli than local motion information.

### 2.3 Discussion

We investigated the relationship between biological motion processing and the perception of global motion coherence.

As expected, biological motion was easiest to discriminate when the stimuli contained both local motion and global form. Disrupting either local motion or global form information significantly reduced the ability to determine the facing direction of the point-light figure. Even though performance on average seemed better for random-position than scrambled stimuli, this difference was not significant. These results differ from previous studies that found better performance for random-position than scrambled point-light figures (Agnew et al., 2016, Pilz et al., 2010, Spencer et al., 2013, Spencer et al., 2016). However, it has to be taken into account that the use of local motion and form information varies depending on the task and stimulus used (Thirkettle et al., 2009).

Interestingly, when correlating performance for all three point-light stimuli, we found a significant positive correlation between performance for discriminating the facing direction of the normal and random-position stimuli, in which predominantly the global form information was available, and a correlation between performance for normal and scrambled stimuli, in which predominantly the local motion information was available. However, the correlation between scrambled and random-position stimuli was not significant, which underlines the involvement of different underlying processing mechanisms for these two kinds of stimuli.

Contrary to our hypothesis, we found no relationship between global motion perception and performance in any of the three biological motion conditions, which indicates that the neural mechanisms underlying the processing of these two stimulus classes do not entirely overlap. One key difference between the two tasks is the noise present in the RDKs. In the biological motion task, all presented dots are stimulus-related, whereas only a fraction of the dots in the global motion task contributes to the global motion direction. Often, tasks comparing performance in biological motion perception and global motion processing assess signal-to-noise ratios for point-light stimuli embedded in noise (e.g., Spencer et al., 2000, Billino et al., 2008, Koldewyn et al., 2010, Koldewyn et al., 2011, Spencer et al., 2013). Therefore, a possible relationship between these two tasks might be related to the ability to extract signal from noise. In Experiment 2, we investigated this hypothesis further. We assessed motion coherence thresholds similar to Experiment 1, but this time, we compared performance to point-light actions that were presented with and without surrounding noise.

## 3 Experiment 2

### 3.1 Methods

#### 3.1.1 Subjects

Participants were recruited from the staff and student population at the University of Aberdeen. Twenty five individuals were recruited, however five were excluded because performance accuracy was below chance. The final sample (n = 20, 5 males) ranged in age from 18 to 26 (M = 21.55, SD = 1.88). All had normal or corrected to normal vision (visual acuity >1 on the ETDRS chart) and gave written informed consent. The experiment was carried out in accordance with the Declaration of Helsinki.

#### 3.1.2 Apparatus

The same apparatus used in Experiment 1 was used in Experiment 2.

#### 3.1.3 Stimuli

##### 3.1.3.1 Motion coherence task

Stimuli were RDKs as described for Experiment 1. Motion coherence was set to 45%, and stimulus duration was set to 400 ms.

##### 3.1.3.2 Biological motion

Here, we used point-light figures from the ‘normal’ condition as used in Experiment 1 that contained both local motion and global form information. In a ‘noise’ condition, the point-light tennis player was embedded within a mask of 20 dots moving in random directions at a speed of 4 deg/s, resembling the average motion of the local dots of the point-light stimulus. Stimulus duration was set to 400 ms. In a no-noise condition, the stimulus duration was set to 200 ms. The different durations were chosen because pre-tests indicated that shorter durations in the noise condition would have resulted in floor performance, and longer durations in the no-noise condition resulted in ceiling performance.

#### 3.1.4 Procedure

The procedure was the same as that described in Experiment 1. Participants completed 40 trials in both the motion coherence and biological motion (no noise) tasks, and 80 trials in the biological motion (noise) task. Accuracy of performance was measured for each task.

### 3.2 Results

Mean accuracy in the motion coherence task was 80.7% (SD = 10.45).

**Fig. 5:**
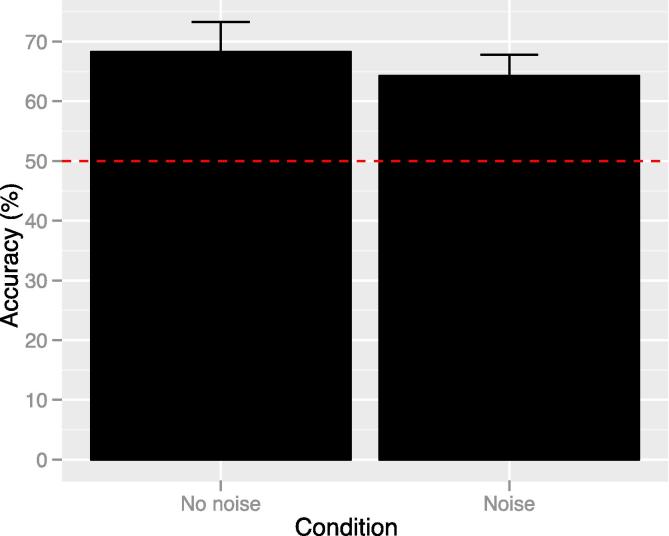
Mean direction discrimination accuracy for biological motion stimuli with and without noise. Errors bars show standard errors of the mean and red line shows chance level. There is no significant difference in accuracy between the two conditions.

Fig. 5 shows mean accuracy in the two biological motion conditions. There was no significant difference in performance between the noise [mean(SD) = 64.25(15.79)] and no-noise [mean(SD) = 68.25(22.66)] condition: t(19) = 0.934, p = .362.

**Fig. 6:**
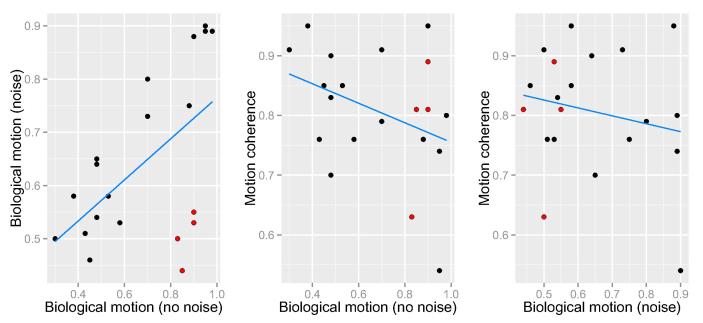
Correlations between proportion accuracy for each biological motion condition and motion coherence (using RDK stimuli). There is a significant correlation between noise and no-noise biological motion (left), but no significant correlation of either biological motion condition with low-level motion coherence (centre and right). Four participants with relatively poor accuracy in the no-noise condition in the biological motion task are highlighted in red. It is clear that their drop in accuracy with the introduction of noise is specific to the biological stimuli. Note that excluding these four participants from analyses does not affect results. (For interpretation of the references to colour in this figure legend, the reader is referred to the web version of this article.)

There was a significant positive correlation between the two biological motion tasks (r_s_ = 0.552, p = .012, 2-tailed). Participants who were better able to determine direction of the action without noise tended to perform well when the action was embedded within noise (see Fig. 6, left). However, there are four potential outliers (marked in red in Fig. 6), for whom this pattern is not apparent, and they show very high performance in the no-noise condition, but conversely low performance in the noise condition.

Overall, performance for biological motion processing, with or without noise, did not significantly correlate with performance for discriminating global motion direction: r_s_ = −0.166, p = .485 and r_s_ = −0.339, p = .144 respectively.

### 3.3 Discussion

Experiment 2 further explored the association between global motion perception and biological motion processing by adding noise to the biological motion stimulus. Regarding biological motion perception, on average, performance was worse when noise was added to the stimulus. However, this difference was not significant. Cutting, Moore, and Morrison (1988) reported that in groups of neurotypical young adults, adding linear translating noise such as used in our study affects performance at stimulus durations of 200 ms but not 800 ms. Therefore, one could have expected performance differences at a stimulus duration of 400 ms. However, it should be noted that stimulus durations for the noise and no-noise condition differed to avoid floor or ceiling effects, and this difference in stimulus duration likely masked performance differences. Performance in both conditions was on average around 65%.

We assessed task relationships by conducting correlations and found a significant positive correlation of performance between the two biological motion tasks. However, similar to Experiment 1, there was no significant correlation between motion coherence and either of the two biological motion conditions. The behaviour of the four possible outliers identified by their poor performance in the biological motion tasks illustrates this null finding, as three out of those four participants performed above average in the motion coherence task. These results nicely illustrate the clear dissociation between global motion processing and biological motion processing.

The pattern of inter-task correlations suggests that the noise in an RDK seems to have a different effect on global motion perception than the noise added to the biological motion stimulus, which further highlights the relative independence of high-level biological and global motion perception.

## 4 General discussion

This study investigated the relationship between global motion perception, and the discrimination of facing direction of biological motion using point-light stimuli. Similar tasks have been used in a number of studies in both healthy young adults, older adults, and clinical populations (e.g. Bertenthal and Pinto, 1994, Pilz et al., 2010, Koldewyn et al., 2011, Kim et al., 2013). However, the exact relationship between global motion and biological motion perception, taking into account the local motion and global form involved in biological motion perception, has so far been unknown.

In Experiment 1 we presented participants with three different types of point-light stimuli: Scrambled walkers that primarily contained local motion, random-position walkers that primarily contained global form, and normal stimuli that contained both local motion and global form information. In addition, we assessed motion coherence thresholds for discriminating translational motion from random-dot kinematograms (RDKs). We found a strong correlation between random-position and normal point-light walkers and a weak correlation between scrambled and normal point-light walkers, which did not survive correction for multiple comparisons. These results suggest that global form information is more relevant for biological motion discrimination than local motion information at least in such facing direction tasks as used in the current study. Previous studies have already pointed out that biological motion can be processed using both the local signals and the global form, and it has been suggested that it depends on the task and specific stimulus used as to which one is more beneficial (Thirkettle et al., 2009). There was no significant correlation between motion coherence and biological motion discrimination in this experiment. In Experiment 2, we compared performance for normal point-light stimuli embedded in noise to that for stimuli without noise, and individual motion coherence thresholds. Again, we found a relationship between performance in both biological motion conditions, but correlations between biological motion stimuli and motion coherence were not significant. It is likely that whereas signal segregation is an early step when processing point-light actions in noise, the processing of the global form of the figure is essential to successfully solve the task.

Taking into account the known neural networks involved in biological and coherent motion perception, it seems surprising that we did not find any correlation between global motion and biological motion perception. Global motion and biological motion are both processed in the dorsal stream, and one area that is particularly important to mention in this context is hMT/V5. Neurons in this area have been shown to be particularly sensitive to global motion perception (Britten et al., 1992), and many other studies, including lesion studies (Vaina, 1994), fMRI (Braddick et al., 2001, Tootell et al., 1995), and MEG studies (Anderson et al., 1996, Aspell et al., 2005) have localised hMT/V5 to be involved in global motion perception in humans. In addition to processing global motion, hMT/V5 has also been shown to be involved in biological motion processing and is activated by both normal and scrambled stimuli (Grossman et al., 2000, Giese and Poggio, 2003, Michels et al., 2005). Based on these results, we would have expected strong correlations between performance in biological motion perception for normal and scrambled actions and motion coherence thresholds. However, even though hMT/V5 seems to be strongly involved in both global motion and biological motion perception, the story seems to be a little more complex.

Studies investigating biological motion perception often subtract activation to normal biological motion from that of scrambled motion, both of which activate hMT/V5. Using such a contrast makes it difficult to exactly localise the area that is involved in biological motion processing. Consequently, hMT/V5 is often not localised using such techniques (Grossman et al., 2000). Alternatively, hMT is localised as a region of interest using global motion stimuli, and then the amount of activation biological motion stimuli elicits in this region is assessed. This constrains the area of exploration to that of global motion perception from RDKs. To more specifically localise areas involved in the processing of different motion stimuli, Howard et al. (1996), used a voxel-based analysis to assess pattern of activation for coherent motion, optic flow and biological motion, which shows large patterns of activation within the region of hMT for all three motion stimuli. Interestingly however, the pattern of activation only partially overlapped, and all three kinds of motion produced their own distinct pattern of activation. Portions of the hMT+/V5 region appear to be involved in the processing of biological motion, independent of their role in the perception of global motion or optic flow. Similarly, Ferri, Kolster, Jastorff, and Orban (2013) investigated overlap between the extrastriate body area and hMT/V5. Even though they found voxels in hMT/V5 that responded both to static human bodies and motion, some voxels were solely sensitive to static body information. Taken together, these results suggest a more heterogeneous activation of hMT+/V5 that has not been fully accounted for by most models of motion perception, and might be related to the missing correlation between global motion and biological motion perception as found in our study.

It has to be noted that the task parameters such as the size of the stimuli and the number of dots presented differed between the biological motion task and the motion coherence task in this study. These differences might have precluded a correlation between the two tasks. However, if stimulus parameters affected performance to such extent, it emphasises the problems arising from using performance differences between those two motion tasks to draw conclusions about differences in motion perception abilities between different populations.

Our results are of particular importance to studies that compare performance for biological motion stimuli to global motion perception in special populations to assess whether deficits in biological motion are related to more general deficits in motion perception. If the processes underlying global motion perception and biological motion perception are as distinct as suggested by our results and the fMRI studies described above, comparisons between global motion perception from random-dot kinematograms and biological motion stimuli should be interpreted with caution.
